# Brilacidin, a host defense peptide mimetic, potentiates ibrexafungerp antifungal activity against the human pathogenic fungus *Aspergillus fumigatus*

**DOI:** 10.1128/spectrum.00888-24

**Published:** 2024-07-09

**Authors:** Thaila Fernanda dos Reis, Camila Diehl, Camila Figueiredo Pinzan, Patrícia Alves de Castro, Gustavo H. Goldman

**Affiliations:** 1 Faculdade de Ciências Farmacêuticas de Ribeirão Preto, Universidade de São Paulo, Ribeirão Preto, Brazil; 2 National Institute of Science and Technology in Human Pathogenic Fungi, São Paulo, Brazil; Institut Pasteur, Paris, France

**Keywords:** *Aspergillus fumigatus*, brilacidin, ibrexafungerp, drug resistance

## Abstract

*Aspergillus fumigatus* is the primary etiological agent of aspergillosis. Here, we show that the host defense peptide mimetic brilacidin (BRI) can potentiate ibrexafungerp (IBX) against clinical isolates of *A. fumigatus*. BRI + IBX can inhibit the growth of *A. fumigatus* voriconazole- and caspofungin-resistant clinical isolates. BRI is a small molecule host defense peptide mimetic that has previously exhibited broad-spectrum immunomodulatory/anti-inflammatory activity against viruses, bacteria, and fungi. *In vitro*, combination of BRI + IBX plays a fungicidal role, increases the fungal cell permeability, decreases the fungal survival in the presence of A549 epithelial cells, and appears as a promising antifungal therapeutic alternative against *A. fumigatus*.

**IMPORTANCE:**

Invasive fungal infections have a high mortality rate causing more deaths annually than tuberculosis or malaria. *Aspergillus fumigatus* causes a series of distinct invasive fungal infections have a high mortality rate causing more deaths annually than tuberculosis or malaria. *A. fumigatus* causes a spectrum of distinct clinical entities named aspergillosis, which the most severe form is the invasive pulmonary aspergillosis. There are few therapeutic options for treating aspergillosis and searching for new antifungal agents against this disease is very important. Here, we present brilacidin (BRI) as a synergizer o fibrexafungerp (IBX) against *A. fumigatus*. BRI is a small molecule host defense peptide mimetic that has previously exhibited broad-spectrum immunomodulatory/anti-inflammatory activity against bacteria and viruses. We propose the combination of BRI and IBX as a new antifungal combinatorial treatment against aspergillosis.

## INTRODUCTION

*Aspergillus fumigatus* is a saprophyte thermotolerant fungus that can cause a series of distinct clinical entities named aspergillosis, which the most severe form is the invasive pulmonary aspergillosis (IPA) (1). Currently, recommended treatment against IPA is based on first-line azole drugs and second-line amphotericin and echinocandins, such as caspofungin (1). Due to the increased emergence of *A. fumigatus* azole-resistant mutants (2), new alternatives are necessary to treat aspergillosis. A recent addition to the antifungal repertoire is the triterpenoid ibrexafungerp (IBX), a semi-synthetic derivative of enfumafungin (3, 4). IBX binds to the 1,3-β-d-glucan synthase, the same target of echinocandins, but in contrast to this class of compounds, it has oral bioavailability (3, 4). Currently, IBX is approved as an oral drug for the treatment of vulvovaginal candidiasis, and it is in the process of approval against invasive candidiasis and aspergillosis (3–5). Here, we describe brilacidin (BRI), a host defense peptide mimetic, as a potentiating agent of IBX against *A. fumigatus*.

**Fig 1:**
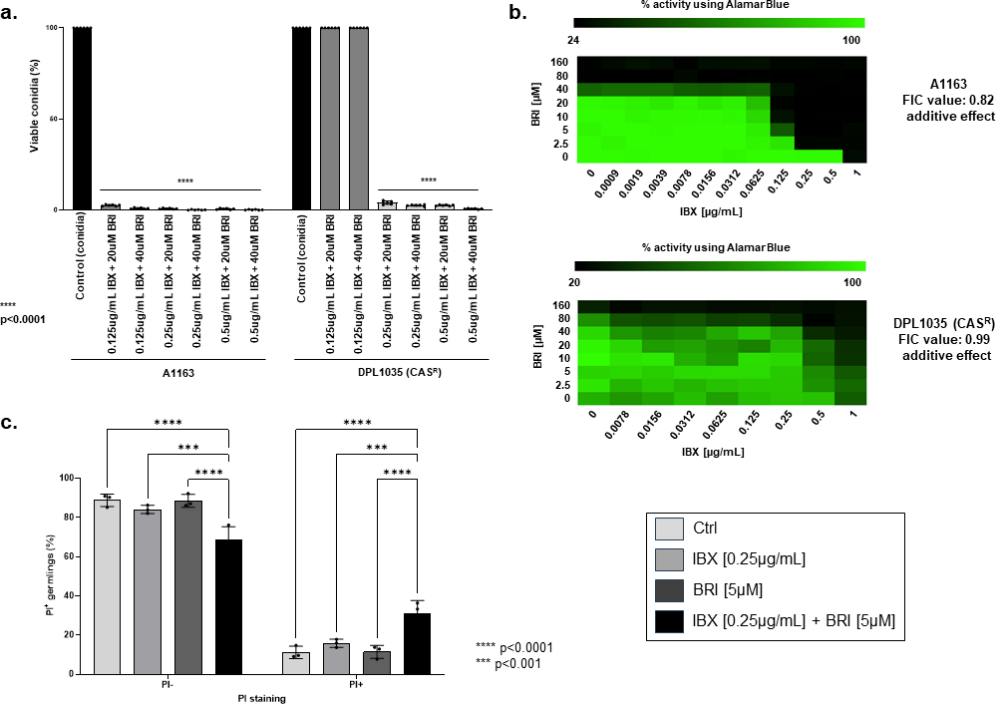
BRI + IBX has synergism against *A. fumigatus*. (a) *A. fumigatus* conidia were incubated for 48 h at 37°C with different combinations of BRI + IBX. After this period, non-germinated conidia were plated on MM, and colony-forming units (CFUs) were assessed. The results are expressed as the % of viable conidia concerning initial inoculum (*n* = 100) and are the average of three repetitions ± SD (*t* test; *P* < 0.0001). (b) The Fractional Inhibitory Concentration (FIC) index for BRI + IBX. Results show the average of three independent experiments. (c) *A. fumigatus* was grown 16 h at 30°C in MM and exposed to MM (control) or MM supplemented with IBX (0.25 µg/mL), BRI (5 µM), or IBX + BRI for 30 min. Propidium iodide (PI) was added (50 µg/mL) for 5 min and the germlings were observed under fluorescent microscope. The results are expressed as the % of PI+ and PI− and show the average ± SD of three independent assays where at least 20 germlings were analyzed each time (*n* ≥ 60; two-way ANOVA with Sidak’s multiple comparisons test; *P* < 0.0001).

BRI is a new chemical entity, a small molecule, constructed aiming to mimic the amphiphilic structure of host defense proteins (HDPs), having one surface with positively charged groups (cationic) and the opposite surface consisting of hydrophobic groups (6–9). With this general synthetic form, there is no need for an agent to be of the size or composition of naturally occurring proteins to effectively function as an HDP, as the ability to act as an HDP is retained by the much smaller synthetic amphiphilic molecule. Previous work showed that the minimal inhibitory concentration (MIC) of BRI for *A. fumigatus* was higher than 80 µM and the combination of 20 µM BRI with 0.2 or 0.5 µg/mL of CAS or 0.125 and 0.25 µg/mL of VOR for 48 h at 37°C reduced and completely inhibited conidial viability between 85% and 100%, respectively, while these concentrations of CAS and VOR alone allowed slow conidial germination (10). To test if BRI could potentiate the effects of another *fks1* (that encodes the 1,3-β-d-glucan synthase) inhibitor, we checked the impact of the combination between BRI and IBX against *A. fumigatus*. A total of 10^3^ conidia of both, the wild-type strain and the CAS^R^ DPL1035, were exposed to different combinations of BRI (20 and 40 µM) and IBX (0.125, 0.250, and 0.50 µg/mL). After 48 h incubation at 37°C, the conidia were plated on MM, and the viability of *A. fumigatus* was assessed by defining the number of colony-forming units (CFUs). Notice that the control for these experiments is the conidial viability (100%) since conidia incubated in the presence of BRI or IBX will grow or be fungistatically inhibited, respectively, making not possible to precisely determine the colony forming units. The different combinations of BRI + IBX reduced conidial viability from 98% to 100% (Fig. 1a). We also observed that a CAS-resistant strain DPL1035 (with a S679P *fks1* mutation) was 100% resistant to combinations of BRI (20 and 40 µM) + IBX (0.125 µg/mL) but showed 96%–100% reduction in conidial viability in the presence of BRI (20 and 40 µM) + IBX (0.25 and 0.5 µg/mL) (Fig. 1a).

To assess the interaction (synergistic, additive, or antagonistic) between the selected compounds and BRI and IBX, checkerboard assays were performed. Briefly, a stock solution of 2.5 × 10^4^ conidia/mL and several dilutions of BRI and IBX were prepared in minimal medium with 10% of alamar blue. In 96-well microtiter plates, IBX was diluted sequentially along the ordinate, while BRI was diluted along the abscissa, to obtain a final volume of 100 µL. The plates were incubated for 48 h at 37°C and the metabolic activity was determined by reading in the spectrophotometer as previously described. Results are expressed as means ± SD from three independent experiments. The Fractional Inhibitory Concentration (FIC) index for BRI + IBX was 0.82 and 0.99 indicating an additive effect against *A. fumigatus* A1163 and DPL1035 CAS-resistant isolate, respectively (Fig. 1b).

Antimicrobial peptides target directly or indirectly the microorganism plasma membrane disrupting their membrane potential (11, 12), and BRI has been shown to act by a similar mechanism in various (non-fungal) microorganisms (13–15). We determined the effect of BRI + IBX on cell permeability by using propidium iodide (PI). This fluorescent DNA-binding dye freely penetrates the cell membranes of dead or dying cells but is excluded from viable cells. When *A. fumigatus* germlings were incubated with IBX 0.25 µg/mL for 30 min, 16.4% were stained by PI, while exposure to BRI 5 µM yielded 10% PI+ (Fig. 1c). The combination of BRI + IBX yielded 35.7% of PI+ germlings (Fig. 1c). These results suggest that, together, BRI + IBX increases the *A. fumigatus* cell membrane permeabilization.

To get more insights about the efficacy of the BRI + IBX combination, we also evaluated if the combination of BRI + IBX could inhibit CAS-resistant and VOR-resistant *A. fumigatus* clinical isolates (Table 1). All these strains, including the reference strain A1163, nine *A*. *fumigatus* clinical isolates VOR-resistant but susceptible to CAS (MEC CAS of 0.25 µg/mL) and two CAS-resistant clinical strains (MEC CAS of 16 µg/mL; strains DPL1035, with a known *fks1* mutation (this strain holds a S679P mutation); and strain CM7555 with unknown mutation(s)] have a MEC of 1.0 µg/mL for IBX and a MIC >80 µM for BRI (Table 1). The addition of BRI at 10 µM combined with 0.25 µg/mL of IBX completely inhibited the growth of all tested strains, including those that are resistant to CAS or with known resistance to azoles (with the TR34/L98H mutation; Table 1). These data suggest that BRI potentiates IBX activity against CAS- or VOR-resistant strains of *A. fumigatus*. Interestingly, one of the *A fumigatus* CAS-resistant mutants that have a mutation in the *fks1* gene is not resistant to IBX (Table 1), suggesting that this mutation at S679P is not able to confer IBX resistance. These results are similar to *Candida* spp. because IBX’s binding site seems to be partially divergent from that of the echinocandins since IBX is active against echinocandin-resistant *Candida* isolates (16, 17).

**TABLE 1:** MECs e MICs of *A. fumigatus* strains

Strains	MEC CAS (µg/mL)	MIC VOR (µg/mL)	MEC IBX (µg/mL)	MIC BRI (µM)	BRI (10 µM) + IBX (0.25 µg/mL)^*a*^	Origin
A1163	0.25	0.5	1.0	>80	−	FGSC^*b*^
DPL1035	16.0	0.5	1.0	>80	−	David Perlin lab
CM7555	16.0	0.5	1.0	>80	−	Gustavo Goldman lab
CYP15-184	0.25	8	1.0	>80	−	Katrien Lagrou lab
CYP15-190	0.25	8	1.0	>80	−	Katrien Lagrou lab
CYP15-192	0.25	4	1.0	>80	−	Katrien Lagrou lab
CYP15-195	0.25	8	1.0	>80	−	Katrien Lagrou lab
CYP15-202	0.25	8	1.0	>80	−	Katrien Lagrou lab
CYP15-212	0.25	8	1.0	>80	−	Katrien Lagrou lab
CYP15-213	0.25	4	1.0	>80	−	Katrien Lagrou lab
CYP15-215	0.25	8	1.0	>80	−	Katrien Lagrou lab
CYP15-220	0.25	4	1.0	>80	−	Katrien Lagrou lab
CYP15-221	0.25	8	1.0	>80	−	Katrien Lagrou lab
CYP15-222	0.25	4	1.0	>80	−	Katrien Lagrou lab
CYP15-224	0.25	8	1.0	>80	−	Katrien Lagrou lab
CYP15-225	0.25	8	1.0	>80	−	Katrien Lagrou lab
CYP15-226	0.25	4	1.0	>80	−	Katrien Lagrou lab
CYP15-228	0.25	4	1.0	>80	−	Katrien Lagrou lab
CYP15-229	0.25	8	1.0	>80	−	Katrien Lagrou lab
CYP15-230	0.25	8	1.0	>80	−	Katrien Lagrou lab
CYP15-231	0.25	4	1.0	>80	−	Katrien Lagrou lab
CYP15-109	0.25	>8	1.0	>80	−	Katrien Lagrou lab
CYP15-147	0.25	8	1.0	>80	−	Katrien Lagrou lab

^*a*^ −, no growth.

^*b*^ FGSC, Fungal Genetics Stock Center (www.fgsc.net).

**Fig 2:**
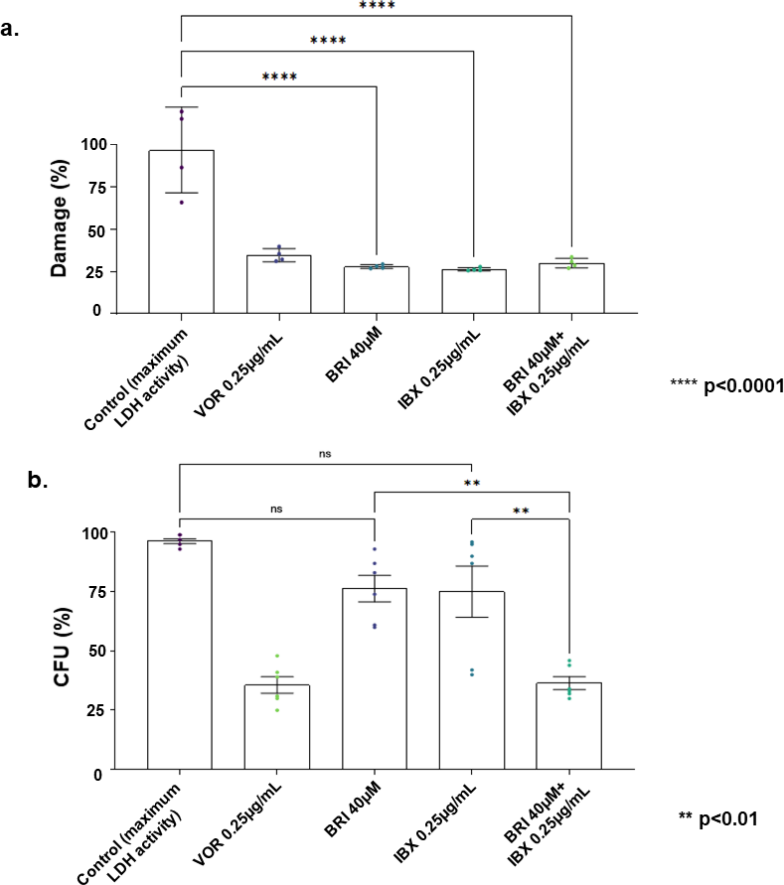
The combination of BRI + IBX is not toxic, causes low level of damage to lung epithelial cells. (a) The cytotoxicity of BRI, IBX, or BRI + IBX was assessed upon exposure of A549 epithelial cells upon 24 h exposure to BRI (40 µM), IBX (0.25 µg/mL), and BRI (40 µM ) + IBX (0.25 µg/mL). Lactate dehydrogenase (LDH) activity was used as a marker for cytotoxicity. The values are expressed as a percentage of cell damage in comparison to the maximum LDH activity obtained after lysing the A549 cells using the lysis buffer reagent provided by the CyQUANT LDH Cytotoxicity Assay kit (Invitrogen). (b) A549 cells were seeded at a density of 10^6^ cells/mL and challenged with *A. fumigatus* conidia at a multiplicity of infection (MOI) of 1:10 in the absence or presence of BRI (40 µM), IBX (0.25 µg/mL), or BRI (40 µM ) + IBX (0.25 µg/mL). After 24 h of incubation, media were removed and the cell suspension was plated on solid Sabouraud Dextrose media. The number of CFUs was determined after 24 h of growth. The CFU percentage for each sample was calculated in comparison with untreated cells (ctrl) and the results were plotted using GraphPad Prism (GraphPad Software, Inc., La Jolla, CA, USA). The fungicidal drug VOR was included as control. All the results are the average of at least three repetitions ± SD. One-way ANOVA was applied as a statistic test; **P value < 0.01; ****P value < 0.0001.

Initially, we determined the toxicity played by BRI + IBX in A549 lung epithelial cells. Toxicity was evaluated by lactate dehydrogenase (LDH) activity and the values are expressed as a percentage of cell damage in comparison to the maximum LDH activity obtained after lysing the A549 cells (Fig. 2a). A549 cells treated with VOR (0.25 µg/mL), BRI (40 µM), IBX (0.25 µg/mL), and BRI + IBX (40µM + 0.25 µg/mL) had between 25% and 30% LHD activity (Fig. 2a). To determine the killing rates of the fungal conidia when exposed to a lung epithelial immortalized cell lineage, the A549 were seeded at a density of 10^6^ cells/mL in 24-well plates. After 24 h, the cells were challenged with conidia at a multiplicity of infection of 1:10 (A549-conidia) and treated with BRI (40 µM), IBX (0.25 µg/mL), and the combination of BRI + IBX (40 µM + 0.25 µg/mL), respectively, at 37°C with 5% (vol/vol) CO2. After 24 h incubation, media were removed and the cells were washed two times with sterile phosphate-buffered saline (PBS). Further, the cell monolayer was scraped away, the cell suspension was collected and diluted in sterile PBS. Then, 100 µL of the diluted sample was plated on Sabouraud agar, the plates were incubated a 37°C overnight and the colonies were counted. To check the fungal viability in the presence of A549 cells, 50 µL of the inoculum was adjusted to 10^3^ /mL and plated on Sabouraud agar to correct CFU counts. The CFU/mL for each sample was calculated and compared to the A1163 reference strain. We observed a significant reduction of more than 25%, 25%, and 65% in the fungal viability in BRI 40 µM, IBX 0.25 µg/mL, and BRI (40 µM) + IBX (0.25 µg/mL), respectively (Fig. 2a). VOR, the standard of care in controlling *A. fumigatus* cell growth, showed a comparable growth reduction to the combination of BRI + IBX (Fig. 2a). Notice that BRI and IBX concentrations used for both experiments were much higher than those observed in Table 1 because we aimed to check for damage to the A549 pulmonary (Fig. 2a) and killing of the conidia (Fig. 2b) by using supra-MIC concentrations.

Taken together, these results indicate that the combination BRI + IBX increases the cell death by increasing the membrane permeability and the fungicidal activity in the presence of A549 lung epithelial cells, and wholly overcomes CAS resistance in echinocandin-resistant *A. fumigatus* clinical isolates and VOR-resistant isolates. Previously, we have demonstrated that combinations of BRI + CAS against *A. fumigatus* affect the cell wall integrity pathway and cell membrane potential (10). Considering that IBX has the same fungal target, that is, the non-competitive inhibition of the β−1,3-glucan synthase activity, it is tempting to speculate that BRI + CAS has a comparable mechanism of action. In summary, our work with BRI + IBX brings an essential contribution to the combination therapies already proposed for IBX, such as IBX + voriconazole, IBX + isavuconazole, and IBX + amphotericin that were synergistic against both *A. fumigatus* azole-susceptible and -resistant strains (18–20). We extended the previously observed number of antifungal drugs BRI can potentiate against *A. fumigatus*.

## Data Availability

Checkerboard data are available from the corresponding author upon request.
